# Impact of Central Asia Stunting Initiative on Stunting and Anemia Among Children and Women of Afghanistan

**DOI:** 10.1111/mcn.70241

**Published:** 2026-10-15

**Authors:** Imran A. Chauhadry, Aminah Jahangir, Muhammad Umer, Khakerah Rashidi, Habibullah Haziq, Rafi Sadeed, Humayun Helal, Muhammad Atif Habib, Imtiaz Hussain, Shabina Ariff, Tatyana Elkour, Sajid B. Soofi, Claudia Hudspeth, Zulfiqar A. Bhuuta

**Affiliations:** ^1^ Centre of Excellence in Women and Child Health Aga Khan University Karachi Pakistan; ^2^ Aga Khan Foundation Geneva Switzerland; ^3^ Aga Khan Foundation Kabul Afghanistan; ^4^ Institute for Global Health and Development Aga Khan University Karachi Pakistan

**Keywords:** Afghanistan, anemia, food insecurity, IYCF, LMIC, nutritious supplementation, pre–post evaluation, stunting, WaSH, women of reproductive age

## Abstract

The Central Asia Stunting Initiative (CASI) is a program designed to address malnutrition among women of reproductive age and children under five in Badakhshan Province, Afghanistan, a region facing significant nutritional challenges. This study used a pre‐post design to assess the impact of CASI interventions, with cross‐sectional surveys conducted at baseline and midline among non‐pregnant women of reproductive age (15–49 years) and children under five using a two‐stage stratified random sampling approach; data collection included anthropometric measurements and hemoglobin assessments. Between the baseline and midline surveys, access to safe drinking water improved from 80.9% to 90.7%, and sanitation improved from 37.4% to 88.3%, while the severe food insecurity rate dropped from 94.7% to 30.5% (*p* < 0.001). Exclusive breastfeeding among children aged 0–5 months increased from 64.0% to 80.9%, and dietary diversity among children 6–24 months rose from 7.5% to 20.7% (both *p* < 0.05). Child under nutrition declined significantly, with underweight decreasing from 35.0% to 15.5% (Cohen's h = 0.46) and stunting from 52.3% to 37.3% (Cohen's h = 0.30). The prevalence of moderate anemia among children fell from 59.7% to 41.1%, while normal hemoglobin levels rose from 39.2% to 58.1% (both *p* < 0.001). Among women, underweight prevalence declined from 20.0% to 9.4% (*p* < 0.001), while overweight prevalence rose from 5.4% to 10.7% (*p* = 0.003). Adjusted models confirmed lower odds of stunting, underweight, and anemia at midline compared with baseline. These findings suggest that CASI was associated with substantial improvements in maternal and child nutrition indicators in Badakhshan, and policymakers should focus on scaling up effective interventions and ensuring continuous monitoring. However, given the single‐group pre‐post design without a comparison group, these findings should be interpreted as temporal associations rather than causal effects.

## Summary

- CASI intervention was associated with reduced child stunting (52.3% → 37.3%) and underweight (35.0% → 15.5%).
- Moderate anemia among children fell sharply, from 59.7% to 41.1%, alongside gains in normal hemoglobin.
- Adjusted models confirm lower odds of stunting, underweight, and anemia at midline versus baseline.
- Findings reflect temporal associations only, given the single‐group pre‐post design without a comparison group.

## 1 Introduction

Malnutrition and micronutrient deficiencies are not just health issues but significant public health crises in developing countries, particularly in Afghanistan (World Health Organization 2024; Kramer and Allen 2015). These challenges are particularly severe for women of reproductive age and children under five (World Health Organization 2024; Kramer and Allen 2015). The complex interplay of socioeconomic vulnerabilities, food insecurity, and limited access to healthcare perpetuates the burden of malnutrition (Katoch 2022; United Nations 2023). Research indicates that undernutrition, poor dietary habits, and insufficient healthcare during pregnancy and lactation create a vicious cycle, adversely affecting women's health and the health and nutrition of children (Wells et al. 2020; Vir 2016; Mamun and Finlay 2015).

Afghanistan is grappling with one of the highest child malnutrition rates globally, with almost 41% of children under five suffering from stunting and 9.5% affected by wasting (UNICEF 2022). Stunting, a chronic form of malnutrition, is predominantly driven by food insecurity, poverty, maternal malnutrition, and inadequate healthcare access (Katoch 2022). It has long‐lasting effects on children's cognitive and physical development, ultimately influencing future productivity and socioeconomic success (Black et al. 2013). Additionally, anemia, the most prevalent micronutrient deficiency, is widespread among Afghan children, affecting nearly 45% of those under five (World Health Organization 2022). Iron deficiency, exacerbated by poor dietary intake, parasitic infections, and inadequate absorption, linked to chronic illnesses, is the leading cause (Mawani et al. 2016). Anemia poses serious risks to child health, leading to delayed development, reduced immunity, and increased morbidity (Bhutta et al. 2013).

Women of reproductive age in Afghanistan experience a dual burden of malnutrition, marked by undernutrition and a growing prevalence of overweight and obesity. Almost 20% of women are underweight, with notable regional differences (Food and Agriculture Organization FAO 2020). Maternal malnutrition is closely tied to adverse birth outcomes, such as low birth weight and neonatal mortality (Martorell and González‐Cossío 1987; King 2016; Nadhiroh et al. 2023). In contrast, the increasing rates of overweight and obesity among urban women accentuate the necessity for comprehensive nutritional strategies that address both sides of the malnutrition spectrum (King 2016). Like children, about 40% of Afghan women of reproductive age suffer from anemia, attributable mainly to iron deficiency, low dietary diversity, and high fertility rates (World Health Organization 2022). Pregnant women are especially at risk, as anemia enhances the likelihood of maternal mortality and adverse pregnancy outcomes (United Nations 2023).

Studies have demonstrated that nutrition interventions based on the 1000‐day approach and supplementation strategies have significantly improved nutrition indicators among women and children (English et al. 2017; Soofi et al. 2022), promising a healthier future generation. The first 1000 days, from conception to a child's second birthday, are a critical window for growth and development, and nutrition interventions during this window have lasting benefits on health and nutrition (English et al. 2017). It has also been recognized that adequate maternal nutrition, achieved through iron and folic acid supplementation, lowers the risk of maternal anemia and low birth weight. At the same time, early initiation of breastfeeding and exclusive breastfeeding contribute to improved child survival and development (Bhutta et al. 2013). Similarly, Vitamin A supplementation, ready‐to‐use therapeutic foods (RUTF), and micronutrient powders have been shown to reduce childhood wasting, stunting, morbidity, and mortality (Bhutta et al. 2013). Evidence suggests that children getting optimal nutrition during this period exhibit better cognitive development, immune function, and long‐term health outcomes (United Nations 2023). Community‐based programs implementing this approach, such as those in South Asia and sub‐Saharan Africa, have successfully lowered malnutrition rates and improved maternal and child health (Soofi et al. 2022; Taneja et al. 2022; Kulwa et al. 2014).

Recognizing the urgency of addressing malnutrition, several organizations have implemented targeted interventions to improve maternal and child nutrition outcomes in Afghanistan. One such initiative is the Central Asia Stunting Initiative (CASI), led by the Aga Khan Foundation (AKF) in collaboration with key stakeholders. The CASI project aims to reduce stunting and improve maternal and child health through community‐based interventions focused on women of reproductive age and children under 5, particularly those under 2 years of age (Table 1). This initiative is being implemented in Badakhshan, a province chosen for its high rates of stunting (58%) and undernutrition (26.1%), making it a crucial location for this study (United Nations Children's Fund UNICEF 2023). This study aims to assess the impact of nutritional interventions in reducing the prevalence of stunting and anemia among Afghan women and children in Badakhshan.

**Table 1:** Interventions implemented under the central asia stunting initiative (CASI).

Target group	Key interventions
Pregnant and lactating women	Antenatal and postnatal care and nutritional assessment Provision of energy‐dense nutritious foods (LNS) Iron and folic acid supplementation Intensive nutrition counseling and behavioral change communication Follow‐ups
Birth to 6 months	Helping Baby Breathe Helping Baby Survive Promotion of exclusive breastfeeding Immunization Behavioral change communication (IYCF practices, care practices, hygiene, and sanitation) Follow‐ups
Children (6–23 months)	Promotion of continued breastfeeding Immunization Promotion of Infant and Young Child Feeding (IYCF) practices Provision of LNS and iron supplementation Growth monitoring Vitamin A supplementation Treatment of acute malnutrition (SAM and MAM) Behavioral change communication (healthy diets, food demonstrations, care practices, hygiene, and sanitation)
Children (24–59 months)	Growth monitoring Improved hygiene and sanitation practices Treatment of acute malnutrition (SAM and MAM) with LNS and RUTF Follow‐ups‐ behavioral change communication (healthy diets, care practices, hygiene, and sanitation)

Beyond program reports, this manuscript presents an independent external evaluation of CASI. We quantify changes using effect sizes (Cohen's *h*), report detailed anemia outcomes alongside anthropometric measures, and provide analyses of maternal nutritional status that have not previously been consolidated for Badakhshan.

## 2 Methodology

The Aga Khan Development Network (AKDN) and its local partners, including the World Food Program and UNICEF, played a pivotal role in delivering the intervention package. CASI implementation in Badakhshan took place between 2019 and 2022; the midline survey was conducted approximately 2 years after program initiation to capture the program's interim impact.

The details of the intervention package are outlined in Table 1. An independent team of researchers from Aga Khan University in Pakistan evaluated the interventions.

Since CASI interventions were administered to a large population in Badakhshan Province, Afghanistan, and given the nature of the intervention, no population segment can be excluded from benefiting; therefore, our study lacks a control group. Given this challenge, we employed a Single‐Group Pre‐Post Test Design to evaluate the effectiveness of the CASI intervention package (Centre for Epidemiology and Evidence 2023). In our study, the participants serve as their own controls, with baseline (pre‐intervention) data compared to mid‐intervention data to assess changes in nutritional indicators.

The survey's evaluation focused on two key groups: non‐pregnant women of reproductive age (15–49 years) and children aged 0–59 months. Using an estimated stunting prevalence of 41% among children under 5 years, a minimum sample size of 350 children was calculated at a 95% confidence level and a 5% margin of error, with an additional 20% allowance for non‐response (Cochran 1963).

Due to security and accessibility constraints, the achieved baseline sample (*N* = 232 children) was smaller than planned. In contrast, the midline survey exceeded the target sample size (*N* = 565 children). The reduced baseline sample size may affect the precision of baseline estimates and the statistical power to detect differences between survey rounds.

The baseline and midline surveys represent independent repeated cross‐sectional samples rather than a longitudinal cohort, meaning that the same individuals were not followed over time. Therefore, observed differences between survey rounds may reflect both true population‐level changes and differences in sample composition.

We employed a two‐stage stratified sampling design, treating villages as primary sampling units (PSUs) and households within those villages as secondary sampling units (SSUs). Villages were selected using proportional probability sampling (PPS), while households were chosen through systematic random sampling. A new household listing was conducted to ensure an accurate selection, prioritizing households with children under the age of five.

Demographics included age, sex, and maternal and paternal literacy (self‐reported reading and writing ability). Socioeconomic status (SES) was derived using an asset‐based wealth index (PCA) constructed from housing materials, water and sanitation, and household assets, then grouped into quintiles. Household food security status was assessed using the Household Food Insecurity Access Scale (HFIAS) categories (food secure, mild, moderate, severe). The Minimum Dietary Diversity (MDD) for children aged 6–24 months was assessed using the WHO Infant and Young Child Feeding (IYCF) indicator, defined as consumption of foods from at least five out of eight food groups in the previous 24 h, based on caregiver recall. Improved Sanitation Facilities were defined as Flush to a piped sewer system or septic tank, Flush to a pit latrine, ventilated improved PIT latrine, and PIT latrine with slab; Improved drinking water sources included piped water, protected wells, protected springs, boreholes, and rainwater collection as defined by the WHO/UNICEF Joint Monitoring Program (JMP).

Improved sanitation facilities included flush or pour‐flush toilets connected to a sewer system, septic tanks or pit latrines, ventilated improved pit latrines, and composting toilets.

The selection of respondents was based on specific criteria. Household heads or knowledgeable members aged 18 years and older provided data on demographics, socioeconomic conditions, and food security. Eligible women in selected households were surveyed for reproductive health and dietary data. Data on children aged 0–59 months were collected from mothers or caretakers, including anthropometric measurements and hemoglobin (Hb) tests on a randomly selected child (6–59 months) per household. In children over 2 years, we calculated height‐for‐age z scores (HAZ); for children under 2 years, we used length instead of height to calculate length‐for‐age z scores (LAZ). We also computed body mass index (BMI) for mothers using height and weight. Children were classified as stunted (height‐for‐age z‐score < −2 SD) or not stunted according to the WHO Child Growth Standards, and mothers were classified as underweight, overweight, or obese, all by the WHO classification, ensuring our survey's alignment with global standards (World Health Organization 2009). Hemoglobin concentration was also adjusted for altitudes above 1000 meters using the WHO‐recommended altitude formula (World Health Organization 2011).

The research and evaluation team of Aga Khan University developed and reviewed a detailed survey protocol. Data collection tools were designed, translated, and pre‐tested to ensure accuracy. The survey was conducted by independent teams from Aga Khan University, trained in data collection, anthropometry, and Hb testing.

Hemoglobin concentration was measured using a portable HemoCue Hb 301 analyzer from capillary blood obtained via finger prick. Field teams were trained in standardized procedures for blood collection and device use. Daily calibration and quality control checks were conducted to ensure accuracy. Hemoglobin values were adjusted for altitude using WHO‐recommended correction factors.

Survey quality was monitored at multiple levels. Field‐based supervision involved team leaders reviewing data collection, anthropometry, and Hb testing. AKU's Data Management Unit (DMU) provided real‐time dashboard tracking and data validation. Regular feedback was given to supervisors to address inconsistencies and enhance data quality.

Data collection utilized Computer‐Assisted Personal Interviews (CAPI) via handheld tablets. Paper‐based personal interviews (PAPI) were conducted in security‐sensitive areas, with data later transcribed into the system. Data entry was performed using a customized Android application with built‐in validation checks, and a web‐based dashboard allowed real‐time monitoring. Data security was ensured through encryption and restricted access.

Measurement and variable definitions were standardized across domains: hemoglobin concentration was measured from capillary finger‐prick blood using a portable HemoCue Hb 301 analyzer with trained field staff, daily calibration, and altitude adjustment per WHO guidelines; child anthropometric indices (height/length‐for‐age, weight‐for‐height, and weight‐for‐age) were calculated using WHO Child Growth Standards, defining stunting, wasting, and underweight as z‐scores < −2 SD, while maternal nutritional status was classified by BMI as underweight (< 18.5 kg/m^2^), normal (18.5–24.9 kg/m^2^), or overweight (≥ 25 kg/m^2^); anemia was defined using WHO thresholds (< 11 g/dL for children 6–59 months and < 12 g/dL for women of reproductive age); infant and young child feeding indicators included minimum dietary diversity (consumption of ≥ 5 of 8 food groups in the previous 24 h among children 6–24 months) and exclusive breastfeeding among infants 0–5 months; water and sanitation were categorized per WHO/UNICEF JMP definitions into improved sources (e.g., piped water, boreholes, protected wells/springs, rainwater) and improved sanitation (e.g., flush toilets, septic systems, ventilated improved pit latrines, composting toilets); and immunization status among children 12–23 months was classified as fully immunized if they received all recommended vaccines (BCG, three doses of pentavalent, three doses of oral polio vaccine, and measles), otherwise partially immunized.

All analyses were conducted using STATA. Given the two‐stage stratified sampling design, the analyses accounted for the complex survey structure using survey‐adjusted procedures, incorporating clustering at the village (primary sampling unit) level and sampling weights derived from probability‐proportional‐to‐size (PPS) selection. Standard errors and 95% confidence intervals were estimated. Descriptive statistics were calculated separately for baseline and midline surveys. Differences between survey rounds were assessed using survey‐adjusted chi‐square tests for categorical variables and survey‐adjusted *t*‐tests for continuous variables. Multivariable logistic regression analyses were conducted using survey‐adjusted models to estimate associations between survey round and key outcomes while adjusting for potential confounders. Covariates included in the adjusted models were selected a priori based on established determinants of child and maternal nutrition, including child age, child sex, maternal education, household wealth quintile, household food security status, and access to improved water and sanitation facilities.

In addition to statistical significance testing, the magnitude of changes in proportions between baseline and midline was quantified using Cohen's *h* (Sullivan and Feinn 2012), calculated as the difference between two arcsine‐transformed proportions. Cohen's *h* values were interpreted using conventional thresholds (0.20 = small, 0.50 = moderate, 0.80 = large). These effect size estimates are unadjusted measures of change and were used to complement, rather than replace, adjusted analyses.

Written informed consent was obtained from all adult participants prior to data collection. For children, informed consent was obtained from mothers or primary caregivers. Participation in the study was voluntary, and respondents were informed of their right to withdraw at any time without consequence.

All data were collected confidentially, and no personal identifiers were included in the analytical dataset. Data were securely stored and accessed only by authorized members of the research team.

Children identified as having severe malnutrition or illness were referred to health facilities for treatment.

### 2.1 Ethics Statement

Ethics approval was obtained from the Aga Khan University Ethics Review Committee (ERC# 2019‐1582‐4219 2019‐07‐03) and the Afghanistan National Public Health Institute (IRB Code: IRB.C.0519.0029).

## 3 Results

In our study, Confidence intervals are presented alongside all key estimates to reflect the precision of measurements. While the baseline sample size was smaller than planned, resulting in wider confidence intervals for some indicators, the direction and magnitude of changes between baseline and midline were generally consistent across multiple outcomes.

Our Results in Table 2 show the baseline sociodemographic and health indicators for the baseline and midline surveys. The findings indicate changes in socioeconomic conditions between baseline and midline survey**s**, characterized by increased access to safe drinking water (from 80.9% to 90.7%) and sanitation facilities (from 37.4% to 88.3%). Food security improved markedly, with severe food insecurity dropping from 94.7% to 30.5%. The data indicate that most mothers fall within the 20–34 age group, with little change in the proportion between baseline (74.2%) and midline (73.5%). The proportion of mothers reported as literate was higher in the midline survey (69.8%) compared with the baseline survey (63.2%), while the proportion of employed mothers was lower at midline (6.8%) than at baseline (9.5%). Similarly, paternal literacy was slightly lower at midline (74.8%) compared with baseline (76.7%).

**Table 2:** Sociodemographic and health indicators, baseline and midline surveys.

Variable	Baseline % (95% CIs)	Midline % (95% CIs)
Household‐level indicators	N = 369	N = 371
Household Size; mean (95% CIs)	8.8 (8.4, 9.3)	8.4 (8.0, 8.8)
SES Status		
Poorest	20.3% (16.2%, 24.4%)	18.6% (14.4%, 22.9%)
Poor	19.9% (15.7%, 24.1%)	19.4% (14.9%, 24.0%)
Middle	19.9% (15.6%, 24.3%)	18.2% (13.9%, 22.6%)
Rich	20.0% (15.6%, 24.5%)	19.7% (15.0%, 24.3%)
Richest	19.8% (15.5%, 24.2%)	24.0% (18.4%, 29.6%)
Type of housing		
Flooring		
Natural	96.1% (94.1%, 98.2%)	74.8% (69.3%, 80.3%)
Rudiment	3.4% (1.5%, 5.3%)	1.2% (0.0%, 2.4%)
Finished	0.5% (0.0%, 1.1%)	24.0% (18.5%, 29.5%)
Roof		
Natural roofing	0.6% (−0.1%, 1.4%)	13.3% (9.6%, 17.0%)
Rudimentary roofing	78.5% (74.3%, 82.7%)	27.4% (22.3%, 32.5%)
Finished roofing	20.9% (16.7%, 25.0%)	59.3% (53.6%, 65.0%)
Walls		
Natural walls	48.2% (42.8%, 53.6%)	32.9% (27.7%, 38.2%)
Rudimentary walls	48.6% (43.3%, 54.0%)	42.1% (36.2%, 47.9%)
Finished walls	3.2% (1.4%, 5.0%)	24.6% (19.0%, 30.2%)
Other	—	0.4% (0.0%, 1.1%)
Access to Safe Drinking Water (Improved sources of water)	80.9% (77.0%, 84.9%)	90.7% (87.8%, 93.6%)
Improved Sanitation Facilities	37.4% (32.2%, 42.6%)	88.3% (84.6%, 92.0%)
Food Security Status		
Food Secure	1.5% (0.2%, 2.9%)	9.1% (5.1%, 13.1%)
Mild Food Insecure	0.4% (0.0%, 1.1%)	25.5% (20.3%, 30.8%)
Moderate Food Insecure	3.3% (1.3%, 5.2%)	34.9% (29.2%, 40.6%)
Severely Food Insecure	94.7% (92.3%, 97.2%)	30.5% (25.4%, 35.6%)
Mothers level indicators	N = 229	N = 541
Mother's Age Group		
Less than 20 years	2.4% (0.8%, 4.1%)	2.4% (0.4%, 4.4%)
20–34 years	74.2% (67.9%, 80.5%)	73.5% (69.2%, 77.9%)
35–49 years	23.4% (17.2%, 29.5%)	24.0% (19.9%, 28.2%)
Mother's Education Level		
Illiterate	36.8% (30.1%, 43.6%)	30.2% (25.9%, 34.5%)
Literate	63.2% (56.4%, 69.9%)	69.8% (65.5%, 74.1%)
Employment Status of Mother		
Working	9.5% (5.2%, 13.8%)	6.8% (4.3%, 9.2%)
Not working	90.5% (86.2%, 94.8%)	93.2% (90.8%, 95.7%)
Father's Education Level	N = 223	N = 544
Illiterate	23.3% (17.5%, 29.1%)	25.2% (21.2%, 29.2%)
Literate	76.7% (70.9%, 82.5%)	74.8% (70.8%, 78.8%)
Child level indicators	N = 232	N = 565
Child's Sex		
Male	49.1% (42.0%, 56.2%)	48.1% (43.3%, 52.8%)
Female	50.9% (43.8%, 58.0%)	51.9% (47.2%, 56.7%)
Child's Age Group		
0–5 months	9.5% (5.1%, 13.8%)	10.0% (7.0%, 13.0%)
6–11 months	9.4% (5.5%, 13.3%)	8.7% (6.1%, 11.3%)
12–23 months	17.6% (12.2%, 23.0%)	24.2% (20.0%, 28.5%)
24–35 months	25.6% (19.4%, 31.7%)	21.7% (17.9%, 25.6%)
36–47 months	21.0% (15.3%, 26.8%)	16.4% (13.0%, 19.8%)
48–59 months	16.9% (11.6%, 22.1%)	18.9% (15.3%, 22.5%)
Exclusively breastfed: Children aged 0–5 months	N = 16	N = 51
	64.0% (36.5%, 91.4%)	80.9% (67.0%, 94.7%)
MDD (6–24 months) IYCF	N = 66	N = 186
	7.5% (1.9%, 13.2%)	20.7% (13.6%, 27.8%)
Immunization status of child (12–23 months of age)	N = 40	N = 136
Not immunized	—	—
Partially immunized	4.4% (0.0%, 10.6%)	10.6% (4.8%, 16.5%)
Fully immunized	95.6% (89.4%, 100.0%)	89.4% (83.5%, 95.2%)

The child‐level indicators reflect key changes between baseline and midline surveys. The gender distribution remained balanced, with a slight increase in the proportion of female children (50.9%–51.9%). Exclusive breastfeeding among children aged 0–5 months increased significantly, rising from 64.0% to 80.9%. Dietary diversity (MDD) for children aged 6–24 months also improved substantially, increasing from 7.5% to 20.7%. While the proportion of fully immunized children aged 12–23 months declined slightly from 95.6% to 89.4%, the rate of partially immunized children increased from 4.4% to 10.6%. These results indicate higher reported breastfeeding rates and improved dietary diversity at midline compared with baseline, with some concern about the slight decline in full immunization coverage.

The Chi‐square tests (Table 3) compared baseline and midline distributions for household‐, maternal‐, paternal‐, and child‐level indicators. Significant results (*p* < 0.05) indicate that the observed changes between baseline and midline are unlikely due to chance. For example, flooring, roofing, walls, sanitation, food security, and several child nutrition indicators showed highly significant differences, reflecting substantial improvements over time. In contrast, variables such as SES status, mother's age group, father's education, and child's sex did not differ significantly (*p* > 0.05), suggesting stability across survey rounds. Overall, the Chi‐Square results highlight meaningful changes in living conditions and health practices while identifying indicators that remained consistent.

**Table 3:** Chi‐square test results baseline and midline surveys.

Variable	Chi‐square	*p* value
SES Status	2.01	0.7341
Flooring	97.31	0.0000
Roofing	202.32	0.0000
Walls	72.62	0.0000
Safe drinking water	13.05	0.0003
Sanitation	204.27	0.0000
Food security	329.27	0.0000
Mother's age group	0.06	0.9722
Mother's education	2.88	0.0899
Mother's employment	1.37	0.2413
Father's education	0.20	0.6512
Child's sex	0.03	0.8590
Child's age group	6.71	0.2429
Exclusive BF (0–5 m)	1.27	0.2591
MDD (6–24 m)	5.17	0.0230
Immunization (12–23 m)	0.51	0.4771

The findings demonstrate significant improvements in children's nutritional status between the baseline and midline surveys (Table 4), with notable reductions in malnutrition and moderate‐to‐large effect sizes, as indicated by Cohen's *h*. The prevalence of underweight children dropped markedly from 35.0% to 15.5%, a significant 19.5% reduction (*p* < 0.001; Cohen's *h* = 0.46; moderate effect size). Severe underweight declined significantly from 13.9% to 2.9% (−11.0%, *p* < 0.001, Cohen's *h* = 0.42, moderate effect size). Stunting decreased from 52.3% to 37.3% (−15.0%, *p* = 0.001, Cohen's *h* = 0.30, moderate effect size), while severe stunting fell from 26.9% to 15.5% (−11.4%, *p* = 0.002, Cohen's *h* = 0.28, small‐to‐moderate effect size). Acute malnutrition (wasting) declined significantly from 11.8% to 3.1% (−8.7%, *p* = 0.001, Cohen's h = 0.35, moderate effect size), although the reduction in severe wasting from 4.2% to 1.2% (−3.0%) was not statistically significant (*p* = 0.067, Cohen's h = 0.19, small effect size). Concurrent stunting and wasting, a critical indicator of combined chronic and acute malnutrition, dropped substantially from 6.6% to 0.5% (−6.1%, *p* = 0.001, Cohen's *h* = 0.38, moderate effect size). Effect sizes (Cohen's *h*) are presented to describe the magnitude of differences between baseline and midline proportions; however, these estimates are unadjusted and should be interpreted alongside regression‐adjusted results.

**Table 4:** Difference in nutrition status among children, *p* values, and effect size baseline versus midline surveys.

	Baseline % (95% CIs)	N	Midline % (95% CIs)	N	Midline‐baseline % diff (95% CIs)	*p* value	Effect size (Cohen's h)
Nutritional status							
Underweight	35.0% (28.5%, 41.5%)	250	15.5% (11.4%, 19.6%)	494	−19.5% (−27.2%, −11.8%)	< 0.001	0.46
Severely underweight	13.9% (9.1%, 18.7%)	250	2.9% (0.8%, 5.0%)	493	−11.0% (−16.3%, −5.8%)	< 0.001	0.42
Stunted	52.3% (45.2%, 59.5%)	228	37.3% (32.3%, 42.3%)	491	−15.0% (−23.8%, −6.3%)	0.001	0.30
Severely stunted	26.9% (20.6%, 33.2%)	228	15.5% (11.8%, 19.3%)	491	−11.4% (−18.7%, −4.1%)	0.002	0.28
Wasted	11.8% (7.3%, 16.4%)	240	3.1% (1.2%, 5.0%)	488	−8.7% (−13.7%, −3.8%)	0.001	0.35
Severely wasted	4.2% (1.4%, 7.1%)	240	1.2% (−0.2%, 2.7%)	488	−3.0% (−6.2%, 0.2%)	0.067	0.19
Concurrent stunting & wasting	6.6% (2.9%, 10.2%)	225	0.5% (−0.2%, 1.1%)	487	−6.1% (−9.8%, −2.4%)	0.001	0.38

Table 5 highlights significant improvements in anemia status among children between the baseline and midline surveys, with notable reductions in moderate anemia and increases in normal hemoglobin levels. There was no evidence of a meaningful change in severe anemia (< 7 g/dL) between baseline and midline surveys, indicating limited progress in addressing the most critical cases. However, moderate anemia (7–10.99 gm/dL) decreased significantly from 59.7% to 41.1%, reflecting an 18.6% reduction (*p* = 0.001, Cohen's *h* = 0.37). Furthermore, the proportion of children with normal hemoglobin levels (≥ 11 gm/dL) increased significantly from 39.2% to 58.1%, representing an 18.9% improvement (*p* < 0.001, Cohen's *h* = 0.38).

**Table 5:** Difference in anemia status among children, *p* values, and effect size baseline versus midline surveys.

	Baseline % (95% CIs)	Midline % (95% CIs)	Midline‐baseline (% diff) (95% CIs)	*p* value	Effect size (Cohen's *h*)
Anemia	**N** = **163**	**N** = **317**			
Severe anemia (< 7 gm/dL)	1.1% (−1.0%, 3.1%)	0.8% (−0.1%, 1.8%)	−0.3% (−2.5%, 2.0%)	0.828	0.03
Moderate anemia (7–10.99 gm/dL)	59.7% (51.4%, 68.1%)	41.1% (34.8%, 47.4%)	−18.6% (−29.1%, −8.1%)	0.001	0.37
Normal (> = 11 gm/dL)	39.2% (30.9%, 47.5%)	58.1% (51.8%, 64.4%)	18.9% (8.4%, 29.3%)	< 0.001	0.38

Table 6 reveals significant changes in the nutritional status of women of reproductive age (WRA) between baseline and midline surveys. The prevalence of underweight women (BMI < 18.5) decreased significantly from 20.0% to 9.4%, a 10.6% reduction (*p* < 0.001, Cohen's *h* = 0.30), indicating notable improvements in nutritional outcomes. Although the proportion of women with a normal BMI (18.5–24.9) increased from 74.1% to 78.3%, this 4.2% rise was not statistically significant (*p* = 0.139, Cohen's *h* = 0.10). Meanwhile, the prevalence of overweight women (25.0–29.9 BMI) rose significantly from 5.4% to 10.7%, reflecting a 5.2% increase (*p* = 0.003, Cohen's *h* = 0.20), and obesity (> 30 BMI) increased from 0.1% to 1.0% (*p* = 0.028, Cohen's *h* = 0.14). These trends suggest an emerging concern for overnutrition. The proportion of missing or unknown data increased slightly from 0.3% to 0.7%, but this change was not statistically significant (*p* = 0.434, Cohen's *h* = 0.06).

**Table 6:** Difference in nutrition status among WRA, *p* values, and effect size baseline versus midline surveys.

	Baseline % (95% CIs)	Midline % (95% CIs)	Midline‐baseline (% diff) (95% CIs)	*p* value	Effect size (Cohen's *h*)
Nutritional status of WRA (15–49 years)	**N** = **472**	**N** = **631**			
Underweight (< 18.5)	20.0% (16.2%, 23.9%)	9.4% (6.9%, 11.9%)	−10.6% (−15.2%, −6.1%)	< 0.001	0.30
Normal (18.5–24.9)	74.1% (69.9%, 78.3%)	78.3% (74.7%, 81.8%)	4.2% (−1.4%, 9.7%)	0.139	0.10
Overweight (25.0–29.9)	5.4% (3.2%, 7.7%)	10.7% (8.0%, 13.4%)	5.2% (1.8%, 8.7%)	0.003	0.20
Obese (> 30)	0.1% (0.0%, 0.3%)	1.0% (0.2%, 1.7%)	0.8% (0.1%, 1.6%)	0.028	0.14

Table 7 shows significant changes in hemoglobin levels among participants between the baseline and midline surveys, reflecting improvements in overall health and nutritional status. The prevalence of severe anemia (< 7 gm/dL) showed a slight change of 0.3%, with no statistically significant difference (*p* = 0.943, Cohen's *h* = 0.00). Moderate anemia (7–11.99 gm/dL) decreased significantly from 32.8% to 22.2%, representing a 10.6% reduction (−10.6%, *p* = 0.003, Cohen's *h* = 0.24), indicating a small‐to‐moderate positive effect of interventions. The proportion of individuals with normal hemoglobin levels (≥ 12 gm/dL) increased significantly from 66.9% to 77.5%, reflecting a 10.6% improvement (*p* = 0.003, Cohen's *h* = 0.24).

**Table 7:** Difference in anemia status among WRA, *p* values, and effect size baseline versus midline surveys.

	Baseline % (95% CIs)	Midline % (95% CIs)			
Hemoglobin level	**N** = **335**	**N** = **431**	Midline‐baseline (% diff) (95% CIs)	*p* value	Effect size (Cohen's *h*)
Severe anemia (< 7 gm/dL)	0.3% (−0.3%, 0.9%)	0.3% (−0.2%, 0.9%)	0.0% (−0.8%, 0.8%)	0.943	0.00
Moderate anemia (7–11.99 gm/dL)	32.8% (27.5%, 38.2%)	22.2% (17.9%, 26.6%)	−10.6% (−17.5%, −3.7%)	0.003	0.24
Normal (> = 12 gm/dL)	66.9% (61.5%, 72.2%)	77.5% (73.1%, 81.8%)	10.6% (3.7%, 17.5%)	0.003	0.24

Multivariable logistic regression analyses were conducted to examine associations between survey rounds and key nutritional outcomes after adjusting for demographic, socioeconomic, and household‐level factors (Table 8). Compared with the baseline survey, children assessed at midline had significantly lower odds of stunting (AOR = 0.58, 95% CI: 0.40–0.84), underweight (AOR = 0.52, 95% CI: 0.35–0.77), and anemia (AOR = 0.61, 95% CI: 0.43–0.86). Similarly, women surveyed at midline had lower odds of being underweight compared with baseline (AOR = 0.46, 95% CI: 0.29–0.73).

**Table 8:** Multivariable logistic regression analysis of nutritional outcomes.

Variable	Child stunting (AOR, 95% CI)	Child underweight (AOR, 95% CI)	Child anemia (AOR, 95% CI)	Maternal underweight (AOR, 95% CI)
Midline vs baseline	0.58 (0.40–0.84)	0.52 (0.35–0.77)	0.61 (0.43–0.86)	0.46 (0.29–0.73)
Child age (months)	1.02 (1.01–1.03)	1.01 (1.00–1.02)	0.97 (0.95–0.99)	—
Female child	0.94 (0.70–1.25)	0.90 (0.68–1.20)	1.05 (0.78–1.42)	—
Maternal education	0.73 (0.54–0.98)	0.70 (0.52–0.95)	0.75 (0.57–0.99)	0.72 (0.54–0.95)
Improved sanitation	0.65 (0.47–0.91)	0.60 (0.42–0.86)	0.70 (0.52–0.95)	0.66 (0.47–0.92)
Improved drinking water	0.89 (0.61–1.29)	0.85 (0.58–1.24)	0.91 (0.65–1.27)	0.88 (0.60–1.30)
Food security	0.71 (0.55–0.92)	0.68 (0.50–0.91)	0.67 (0.50–0.89)	0.63 (0.48–0.82)
Wealth quintile	0.62 (0.41–0.95)	0.60 (0.39–0.92)	0.68 (0.44–1.04)	0.70 (0.45–1.08)

Maternal education, improved sanitation, and household food security were consistently associated with lower odds of adverse nutritional outcomes across models. For example, maternal literacy was associated with reduced odds of child stunting (AOR = 0.73, 95% CI: 0.54–0.98) and maternal underweight (AOR = 0.72, 95% CI: 0.54–0.95). Access to improved sanitation was also associated with lower odds of stunting (AOR = 0.65, 95% CI: 0.47–0.91) and underweight (AOR = 0.60, 95% CI: 0.42–0.86). In contrast, improved drinking water was not significantly associated with nutritional outcomes after adjustment.

Increasing child age was associated with slightly higher odds of stunting and underweight but lower odds of anemia, while child sex was not significantly associated with any of the outcomes. Higher household wealth was associated with lower odds of child stunting and underweight, although associations with anemia and maternal underweight were not statistically significant.

Overall, the results demonstrate the effectiveness of interventions to address undernutrition and anemia, mainly through targeted nutritional programs. However, the findings also underscore the need for sustained efforts to tackle severe cases of malnutrition and anemia and to address the growing prevalence of overweight and obesity, emphasizing the importance of balanced and holistic health interventions.

## 4 Discussion

This study examined changes in maternal and child nutrition indicators during the implementation period of the CASI in Badakhshan Province. Substantial improvements were observed across several indicators, including reductions in child stunting, wasting, underweight, and anemia, as well as improvements in household‐level determinants such as sanitation and food security. However, given the study design, these findings should be interpreted as temporal associations rather than causal effects. The reductions in stunting, wasting, and underweight prevalence, as well as in anemia among children, along with improvements in maternal nutritional indicators, highlight the effectiveness of the intervention package. The improvements observed are consistent with findings from other settings where integrated nutrition interventions have been implemented. The substantial decline in severe food insecurity from 94.7% to 30.5% observed between surveys may reflect improvements in household conditions during the study period, although these changes cannot be attributed solely to the intervention (UNICEF 2023). The improvement in literacy rates among mothers from 63.2% to 69.8% may have contributed to better nutrition practices, as maternal education is a known determinant of child health outcomes (Katoch 2022). Furthermore, the increase in exclusive breastfeeding rates from 64.0% to 80.9% is encouraging, as it is associated with reduced child mortality and improved immune system function (Black et al. 2013).

Several alternative explanations may account for the observed changes. Improvements in food security and sanitation may reflect broader contextual changes, including concurrent humanitarian assistance programs, national policy initiatives, or shifts in economic conditions. Additionally, Afghanistan experienced substantial political and health system changes during the study period, which may have influenced access to services and health outcomes. The observed changes may also partially reflect regression to the mean or differences in sample composition between baseline and midline surveys. Therefore, the findings should be interpreted with caution.

Despite these improvements, specific challenges remain. The decrease in the proportion of fully immunized children from 95.6% to 89.4% raises concerns about the sustainability of immunization efforts and potential setbacks in child health protection. Similarly, while anemia prevalence in children decreased significantly, severe cases remained unchanged, indicating a need for more intensive anemia control measures, such as iron‐folic acid supplementation and deworming (Mawani et al. 2016). The potential causal interactions between CASI interventions and observed improvements can be explored through various pathways. Improved maternal nutrition and dietary diversity contribute to better birth outcomes, reducing neonatal morbidity and improving child growth (Bhutta et al. 2013). Increased access to fortified foods and dietary supplementation directly improves micronutrient intake, thereby decreasing anemia and supporting cognitive and physical development (WHO 2024). Community education and awareness programs enhance knowledge on appropriate infant and young child feeding practices, leading to improved exclusive breastfeeding rates and better child nutritional status (UNICEF 2023). Moreover, improved sanitation and hygiene reduce exposure to infections, which can exacerbate malnutrition (Wells et al. 2020).

The observed decline in full immunization (with a corresponding rise in partial immunization) likely reflects external health system disruptions and access barriers during the study period (e.g., service interruptions, supply chain issues, conflict‐related mobility constraints) rather than any adverse effect of CASI. CASI primarily targeted nutrition behaviors and services rather than vaccination delivery.

The literature shows that our findings on the positive impact of CASI interventions are consistent with similar programs implemented in neighboring countries. In Pakistan, the Lady Health Worker (LHW) program and the Benazir Income Support Program (BISP) have contributed to a reduction in stunting rates, from 45% in 2012 to 37.6% in 2019 (29). The success of Pakistan's programs is attributed to integrated maternal and child health initiatives, cash transfers, and improved access to healthcare (Soofi et al. 2022). India's Integrated Child Development Services (ICDS) program and the National Nutrition Mission (POSHAN Abhiyaan) have led to significant reductions in child malnutrition (Singh et al. 2024). Technology‐driven monitoring and maternal health counseling have been crucial in these improvements. Furthermore, micronutrient supplementation programs, including flour fortification with iron and folic acid, have helped reduce the prevalence of anemia among women in many developing countries (Pachón et al. 2015). Community nutrition education and better access to primary healthcare have also contributed to improving child growth indicators. These comparisons highlight that while Afghanistan has made commendable progress through CASI, sustained efforts and policy reinforcement are necessary to achieve long‐term improvements, such as those observed in neighboring countries.

While the findings of this study are valuable, several limitations must be acknowledged. The study used a single‐group pre‐post‐test design, which limits the ability to establish causality. The use of a single‐group pre–post repeated cross‐sectional design without a comparison group limits the ability to establish causal relationships between the intervention and observed outcomes. Changes observed between baseline and midline surveys may reflect secular trends, concurrent humanitarian or government programs, or broader contextual changes, including political and health system disruptions during the study period. Additionally, regression to the mean and differences in sample composition between survey rounds may contribute to observed differences. These factors represent important threats to internal validity and should be considered when interpreting the findings. Follow‐up assessments are necessary to determine whether nutritional gains are sustained over time. As the data were collected through household surveys, there is a risk of social desirability bias, where respondents may overreport positive health behaviors, such as breastfeeding practices or dietary diversity.

Moreover, the baseline sample size was smaller than originally planned due to security constraints, which may reduce the precision of baseline estimates and widen confidence intervals. This limitation may also affect the statistical power to detect differences between survey rounds and could lead to instability in effect size estimates. In addition, the baseline and midline surveys represent independent cross‐sectional samples rather than a cohort, meaning that differences in sample composition between survey rounds may partially explain observed changes. These factors should be considered when interpreting the findings.

The study focuses on Badakhshan Province, which may not be representative of the country as a whole. Differences in cultural practices, healthcare access, and economic conditions across regions may lead to varied intervention effectiveness. While the study notes an increase in overweight and obesity among women, it does not explore the contributing factors in detail. Future research should investigate dietary patterns, physical activity levels, and metabolic health indicators to develop targeted interventions for addressing the dual burden of malnutrition.

Additionally, we did not include biochemical markers (e.g., serum ferritin, folate, vitamin A) or dietary intake assessments to more directly evaluate ‘actual’ nutritional status; instead, we relied on validated proxies (anthropometry and hemoglobin). Future evaluations should incorporate biochemical and dietary assessments.

## 5 Conclusion

Substantial improvements in maternal and child nutrition indicators were observed during the CASI program's implementation period in Badakhshan Province, Afghanistan, leading to notable reductions in malnutrition. Policymakers should focus on scaling up effective interventions, strengthening food security measures, and ensuring continuous monitoring to improve nutrition outcomes for women and children in Afghanistan. However, due to the study design, causal attribution cannot be established, and the findings should be interpreted as temporal associations.

## Author Contributions

Z.A.B., S.B.S., A.J., and C.H. conceptualized the study. M.U., M.A.H., I.H., A.J., and I.A. contributed to the design, tools, and implementation of the research. I.A.C. was responsible for data analysis and interpretation. M.A.H., I.A.C., A.J., and M.U. developed the first draft of the manuscript. All authors critically reviewed the final version of the manuscript and approved it for publication.

## Conflicts of Interest

The authors declare no conflicts of interest.

## Data Availability

The data that support the findings of this study are available from the corresponding author upon reasonable request.
